# Point clouds segmentation of rapeseed siliques based on sparse-dense point clouds mapping

**DOI:** 10.3389/fpls.2023.1188286

**Published:** 2023-07-14

**Authors:** Yuhui Qiao, Qingxi Liao, Moran Zhang, Binbin Han, Chengli Peng, Zhenhao Huang, Shaodong Wang, Guangsheng Zhou, Shengyong Xu

**Affiliations:** ^1^ College of Engineering, Huazhong Agricultural University, Wuhan, China; ^2^ Key Laboratory of Agricultural Equipment for the Middle and Lower Reaches of the Yangtze River, Ministry of Agriculture, Huazhong Agricultural University, Wuhan, China; ^3^ School of Mathematics and Computer Science, Wuhan Polytechnic University, Wuhan, China; ^4^ School of Geosciences and Info-Physics, Central South University, Changsha, China; ^5^ College of Plant Science & Technology, Huazhong Agricultural University, Wuhan, China

**Keywords:** rapeseed siliques, 3D Reconstruction, sparse-dense point clouds mapping, point clouds segmentation, silique recognition

## Abstract

In this study, we propose a high-throughput and low-cost automatic detection method based on deep learning to replace the inefficient manual counting of rapeseed siliques. First, a video is captured with a smartphone around the rapeseed plants in the silique stage. Feature point detection and matching based on SIFT operators are applied to the extracted video frames, and sparse point clouds are recovered using epipolar geometry and triangulation principles. The depth map is obtained by calculating the disparity of the matched images, and the dense point cloud is fused. The plant model of the whole rapeseed plant in the silique stage is reconstructed based on the structure-from-motion (SfM) algorithm, and the background is removed by using the passthrough filter. The downsampled 3D point cloud data is processed by the DGCNN network, and the point cloud is divided into two categories: sparse rapeseed canopy siliques and rapeseed stems. The sparse canopy siliques are then segmented from the original whole rapeseed siliques point cloud using the sparse-dense point cloud mapping method, which can effectively save running time and improve efficiency. Finally, Euclidean clustering segmentation is performed on the rapeseed canopy siliques, and the RANSAC algorithm is used to perform line segmentation on the connected siliques after clustering, obtaining the three-dimensional spatial position of each silique and counting the number of siliques. The proposed method was applied to identify 1457 siliques from 12 rapeseed plants, and the experimental results showed a recognition accuracy greater than 97.80%. The proposed method achieved good results in rapeseed silique recognition and provided a useful example for the application of deep learning networks in dense 3D point cloud segmentation.

## 1 Introduction

Rapeseed is one of the main oil crops in the world (Shyam et al., 2012). The yield of rapeseed is mainly determined by the number of siliques per plant, the number of seeds per silique, and the thousand seed weight (Tang et al., 2020). Among them, the number of siliques per plant has the highest correlation with yield. However, currently, the counting of rapeseed siliques is still done manually, which is not only time-consuming and laborious but also usually has a high cost. This to some extent has limited the rapid progress of rapeseed-related research. Therefore, the intelligent recognition, segmentation, and counting of rapeseed siliques have important research significance.

The 2D image-based silique counting method is fast in detection and high in throughput (Du et al., 2023). For example, Liu et al. proposed an improved K-means-based method for counting wheat ears. Utilizing the clustering of color features, the number of sub-regions within the clustered region was employed as a mean of approximating the wheat crop count. The accuracy of wheat ear counting reached 94.69% (Liu et al., 2019). Dandrifosse, S proposed a deep learning method. It is an unsupervised learning method using the YOLOv5 model on and the cutting edge DeepMAC segmentation method capable of counting and segmenting wheat ears (Dandrifosse et al., 2022). Wang, proposed an improved efficient entdet-d0 model. The introduction of the convolutional block attention module (CBAM) in the model allows the model to refine the features, focus more on the wheat ears and suppress other useless background information. The problem of overlap in wheat ears images can be solved in wheat ears detection and counting (Wang et al., 2021). Zhang proposed a high-precision wheat head detection model with strong generality based on a single-stage network structure. The number of wheats can be detected quickly (Zhang et al., 2022). Liu et al. accurately calculated the number of canola siliques by scatter treatment and image analysis of piled-up siliques(Ren-feng et al., 2020). However, there are high requirements for automated loading and unloading devices. Zhao proposed a P2PNet-Soy method that maximizes the performance of the model in soybean counting and localization by adjusting the architecture and subsequent post-processing. It achieves higher accuracy than the original P2PNet (Zhao et al., 2023). However, traditional methods for 2D image processing have difficulty in solving the occlusion problem due to the lack of high-level semantic features. The existing counting methods based on deep learning of 2D images are also ineffective in solving the occlusion problem. In contrast, precise 3D point clouds data could obtain from various sensors such as Time-of-Flight (ToF), structured light cameras, and 3D laser scanners, which can provide more detailed spatial information about plants to solve the above problems.

High resolution and accurate 3D point cloud data allow for more accurate 3D morphological parameters of plants (Ni et al., 2021). 3D reconstruction is the basis for 3D three-dimensional phenotyping studies of crops, but the requirements for algorithms are high. For instance, with the Kinect V2 (a kind of camera), Xu et al. captured images of rapeseed branches from four different perspectives to reconstruct the rapeseed branches. Then they used the super-green segmentation algorithm to extract the siliques, removed the largest connected domain using opening operation, and identified and located individual siliques through Euclidean clustering (Shengyong et al., 2019). However, due to the swinging effect caused by the tall and soft rapeseed plants, the measurement errors of ToF and structured light sensors used widely range from millimeter to centimeter level (Tao and Zhou, 2017). Moreover, the slender shape of rapeseed siliques requires high spatial resolution for depiction. Therefore, the 3D data of rapeseed plants obtained by ToF or structured light sensors may be less accurate and reliable. 3D scanners (e.g., laser and radar) will give high-precision point clouds. There are more than 100,000 data points for an entire plant. Each plant organ has more than 100,000 to 30,000 data points. Although the point clouds obtained by this method are of high quality, they suffer from problems such as expensive equipment (Jin et al., 2021). A low-cost and high-precision method to construct 3D point clouds of plants can be achieved using smartphone and SFM algorithm (Marzulli et al., 2020). However, the cumbersome process of taking photos from multiple angles greatly affects the efficiency of acquiring point clouds. Therefore, this study proposes a video-based SFM 3D reconstruction method for an entire mature rapeseed plant, aiming at achieving low-cost, high-accuracy, and high-efficiency 3D reconstruction. This makes it possible to divide and count whole rapeseed siliques with high precision. However, the numerous siliques of the mature rapeseed are scattered and partially overlapping, making it difficult to identify them accurately. Therefore, high-precision identification and segmentation of rapeseed siliques remain a challenge (Li et al., 2022). Although the rapid development of deep learning has facilitated research on plant point cloud segmentation. However, previous research has mainly focused on methods based on hard voxelization or down sampling. These methods are limited to segmenting simple plant organs. Segmentation of complex plant point clouds with high spatial resolution remains challenging. Recent deep learning methods to segment the to point clouds have emerged to address this challenge. They learn features from the input data in a data-driven manner [23]. Thanks to advanced neural networks, deep learning has shown great potential in plant 3D phenotypic analysis, surpassing most traditional segmentation methods (Guo et al., 2020). However, high-precision 3D point clouds places high demands on computer performance, especially graphics cards (Shi et al., 2019). Ghahremani et al. proposed a new pattern-based deep neural network, Pattern-Net, for segmentation of wheat point clouds. For the first time, they partitioned wheat point clouds into defined organs. And their features are analyzed directly in 3D space. The network provides a method for the actual segmentation of plant parts directly in the point cloud domain (Ghahremani et al., 2021). However, the network requires high computer performance and average recognition accuracy. A point-based fully convolutional neural network (PFCN) was proposed by Jin et al. The network directly uses points containing only geometric information. Then extracts point-by-point and block-by-block features to classify each point. The method addresses the difficulty of segmenting large-scale forest scenes (Jin et al., 2020). A bifunctional deep learning neural network (PlantNet) was proposed by Li et al. The method implemented semantic segmentation and instance segmentation of two dicotyledons and one monocotyledon in a point cloud (Li et al., 2022). However, high-precision 3D point cloud processing places high demands on computer performance, especially on the size of graphics memory. Therefore, existing network architectures and hyperparameters are mainly designed for small-scale inputs under current hardware limitations. Down sampling the point clouds before feeding it into a point-based network is necessary, but it also leads to significant loss of original data information.

In summary, the main challenge for segmentation of dense and small sized rapeseed siliques in two reasons. Firstly, both the hard voxelization, which is widely used in voxel-based methods, and the down sampling operation in point-based methods result in a significant loss of information from the original data. Secondly, training and inferring on dense pixel grids or point clouds may impose intolerable computational costs on existing deep learning methods. Therefore, this paper proposes an efficient and high-precision deep learning segmentation method for sparse point clouds that uses DGCNN (Wang et al., 2019) to segment and then maps the results onto dense point clouds. This method accurately identifies and segments rapeseed siliques while maintaining complete spatial information and requiring low computer performance, making it a low-cost and high-efficiency method for silique identification. The method proposed in this paper provides a useful example of deep learning applied to dense 3D point clouds segmentation and lays the foundation for low-cost and high-efficiency intelligent measurement of rapeseed, promoting further research on rapeseed.

## 2 Materials and methods

### 2.1 An overview of the proposed silique identification methods

**Figure 1:**
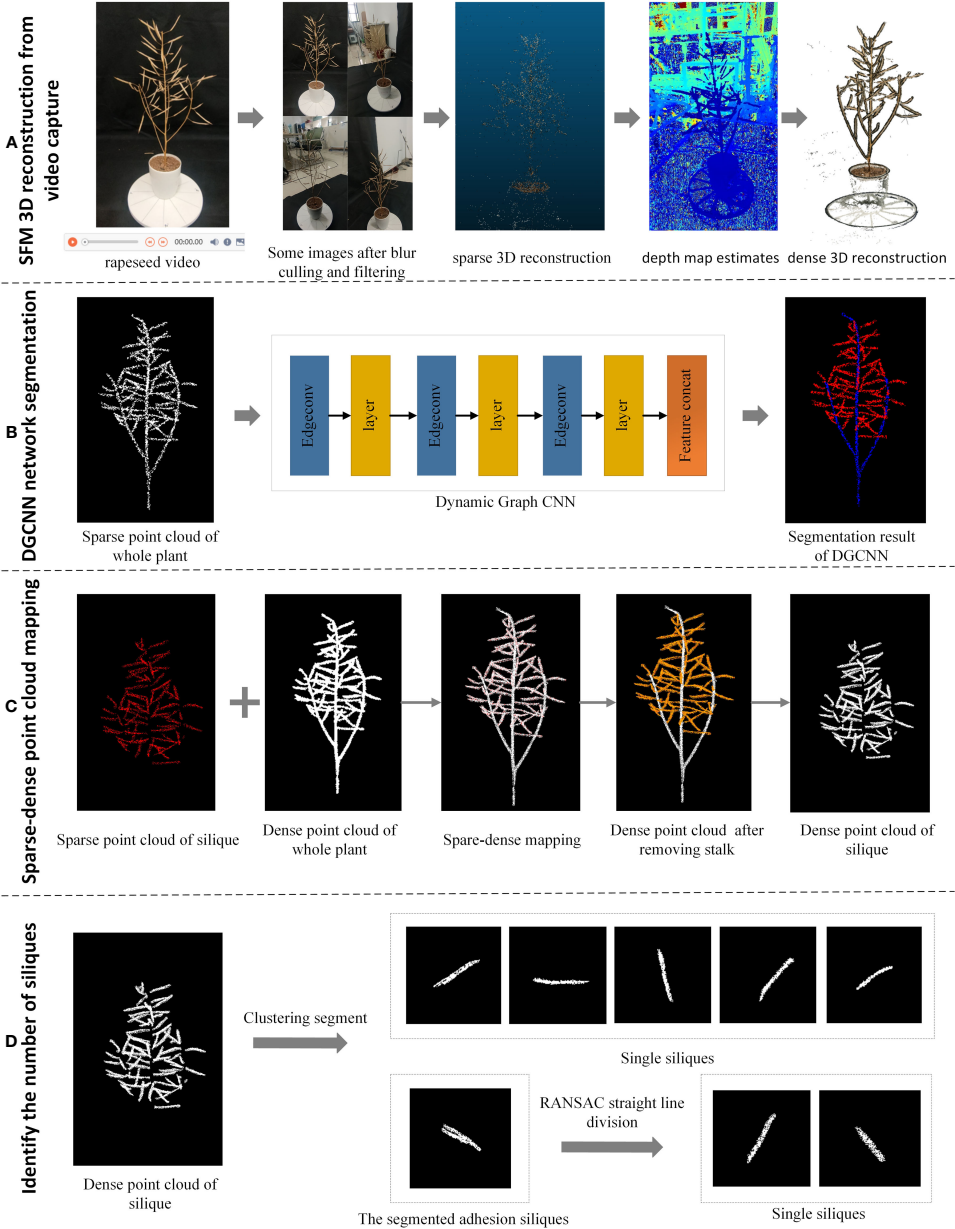
Flow chart of overall silique identification and counting. **(A)** Process for SFM 3D reconstruction from video capture **(B)** Segmentation of canopy silique point clouds after downsampling using the DGCNN network **(C)** A segmentation method using sparse-dense point cloud mapping to obtain the original canopy silique process **(D)** A straight-line fitting approach to segmentation using the Euclidean clustering-RANSAC algorithm to identify siliques process.

First, a circle video was captured around the rapeseed plant using a smartphone. Then the frames are filtered to meet the requirements of high definition, a complete view and no significant redundancy. Some of the over-exposed and dark images are then enhanced to obtain a high-quality video collection to improve the speed and quality of the 3D reconstruction. The processed video frames are then used as input to obtain a 3D dense point cloud after sparse reconstruction, depth estimation, and dense reconstruction (**Figure 1A**).

Next, the whole rapeseed silique point cloud was then obtained using straight pass filtering and down sampling to obtain the target horn fruit point cloud. The DGCNN network segmentation method was then used on this target point cloud. The down-sampled canopy canola rapeseed silique with the stalks removed were obtained (**Figure 1B**).

Then, the sparse-dense point cloud mapping segmentation method was then applied to the down sampled canopy rapeseed silique to obtain the original canopy rapeseed silique with stalks removed (**Figure 1C**).

Finally, Euclidean clustering was used to segment for canopy siliques. For the adherent siliques a linear fitting method based on a random sampling consistency algorithm was used to segment the identified siliques (**Figure 1D**).

The overall flowchart is shown in **Figure 1**.

### 2.2 Experimental materials and data collection

Mature rapeseed plants, whose varieties are Zhongshuang 6, Dadi 55, and Huayouza 62, were collected from experimental fields in Ezhou Base in May 2022. Thirty plants each variety were manually counted for the number of siliques per plant. Image acquisition was conducted indoors under natural light. The entire rapeseed plant was fixed in a flowerpot and photographed using a smartphone. The rapeseed was placed in the center of a device with a black background cloth surrounding it. A Xiaomi 10 smartphone was used to record 4K (3840 x 2160, 30fps) videos, with default imaging mode selected. The distance between the phone and rapeseed siliques was about 1-2 meters, and the video was about 30 seconds long. The distance between the phone and the siliques of the rapeseed depends on the size of the rapeseed. The larger the rapeseed the greater the distance. Simply fill your phone’s viewfinder with the entire rapeseed. Some of the captured video images are shown in **Figure 1A**. The algorithm development and testing platform for this paper was a general-purpose computer (Intel Core 11th generation i9-12900K processor with a frequency of 2.5-5.2 GHz, 64GB memory, NVIDIA GeForce RTX 3090 graphics card with 24GB graphics memory), Windows 10 Professional Edition, VS 2019 + PCL 1.80, and Python 3.6.

### 2.3 3D reconstruction based on video key frames and SFM

Using sequential images, the steps for 3D reconstruction include image data collection and preprocessing, sparse point clouds reconstruction, depth map estimation, and dense point clouds reconstruction. The reconstruction process is shown in **Figure 1A**. SFM method requires capturing 100 images from different angles, which is tedious and inefficient. Using a smartphone to capture short videos is undoubtedly a more efficient and effortless way. However, compared to using a single image sequence, 3D reconstruction, based on video and SFM, requires solving two new problems. Firstly, 3D reconstruction, based on SFM, requires feature point extraction and matching. However, the unavoidable shaking during smartphone shooting can cause image blurring and dragging, making feature point detection difficult. Secondly, SFM algorithm only requires around 100 sequence images. But a 30-second video captured by a smartphone can have 900 frames after decompression. Hence, directly using these images for 3D reconstruction will consume enormous computing power (Pepe et al., 2022). To address these two problems, we propose targeted solutions. On the one hand, for blurred images, Laplacian blurred image rejection based on convolutional variance can eliminate them. On the other hand, for redundant images, we use a similar image filtering method based on feature-matching similarity.

#### 2.3.1 Laplacian blurred image rejection based on convolutional variance

A Laplacian convolution is performed on the image to calculate the variance, which is used to measure the image clarity. The calculation result quantifies the high-frequency information in the image. The larger the numerical value, the more high-frequency information the image contains, corresponding to a clear image; while a smaller numerical value corresponds to a blurry image with lost edge details. The commonly used Laplacian template has the disadvantage of weak anti-noise interference ability, so it needs to be smoothed before convolution. Bilateral filtering can effectively remove noise while preserving edges. In this paper, we choose to first apply bilateral filtering to the image for denoising, then convert the color image to grayscale, and perform Laplacian convolution to obtain the final variance (Rehman et al., 2022). This can be expressed mathematically as:


(1) $$F={\frac{\sum_{i=0}^{I}\sum_{j=0}^{J}\left(P\left(\text{i,j}\right)-\bar{P}\right)}{I*J}}^{2}$$

Where *F* is the sharpness value, *I* and *J* are the length and width of the 2D image, *P*(*i, j*) is the pixel value at a point of the image and *P* is the average pixel value of the 2D image.

#### 2.3.2 Similar image filtering based on feature-matching similarity

To extract a subset of keyframes from a large number of video frames while avoiding including too many redundant images, similarity detection on the images is necessary. The image similarity calculation based on feature point matching can solve this problem. For the same object, even if there is a significant change in angle between two images, our method can still judge the similarity based on the number of matched feature point pairs detected. The results obtained are relatively accurate. Each normal image has a certain number of feature points distributed throughout the image, such as edge points and corner points. Matching the feature points detected from two images, the higher the number of correctly matched pairs, the higher the image similarity.

The first step: Read in the two images to be detected, img1 and img2, and convert them to grayscale images, gray1 and gray2.

The second step: Create a SURF feature extractor and select the fast library for approximate nearest neighbors.

The third step: Extract the image features of gray1 and gray2, namely the feature points and the feature vectors around the feature points.

The fourth step: Perform SURF feature matching on the extracted feature points, obtain the number of matches, Matches, and set an empirical threshold of 0.7 to remove points that do not meet the matching requirements, leaving the number of correctly matched features, MatchNum.

The fifth step: Calculate the ratio of the number of correctly matched feature points to the total number of feature points, which represents the similarity value, denoted by Similarity. The larger the Similarity value, the more similar the images are. The formula for calculating Similarity is:


(2) $$Similarity=\frac{MatchNum*100}{Matches}$$

The process of the video keyframe extraction algorithm is as follows: First, set the sampling interval K according to the video duration. Then take out the first frame image and check if it is a blurry image. If it is blurry, continue to check the next frame until it meets the requirements and is used as the starting frame. Then take out the frame at a distance interval of K and continue to check if it is a blurry image. After it meets the clarity requirements, calculate the similarity between these two frames. If the similarity reaches S1 but does not exceed S2, take out this frame image. If the similarity is higher than S2, detect the (K+I)th frame. If the similarity is lower than S1, detect the (K-I)-th frame until it meets the requirements. Repeat the above steps until all images have been checked.

### 2.4 Point clouds segmentation methods of rapeseed siliques

#### 2.4.1 Deep learning dataset

First, the 3D model of rapeseed siliques was reconstructed based on SFM algorithm. Then, the Cloud Compare open-source toolbox was used to create rapeseed silique point clouds dataset by labeling the stems and canopy siliques of the training data separately. The stem part was labeled as 0, and the canopy siliques were named as 1, and saved as “txt” format. When training the model using deep learning methods, a sufficient number of training sample images are required. Consider the rotational translation invariance and scale invariance of a point cloud. For each point in the point cloud dataset, a random translation vector can be generated in the x, y and z directions. The point coordinates are then added to this vector to achieve a random translation. Each component of the translation vector can be randomly generated between [-0.2, 0.2]. Three scaling factors can also be generated randomly between [0.65, 1.7], corresponding to the scaling factors in the x, y and z directions respectively. The coordinates of the point are then multiplied by each of the three scaling factors. This results in random anisotropic scaling to augment the training data. A total of 90 sets of training datasets were created, which expanded the dataset to six times its original size. In total, 540 sets of rapeseed siliques point clouds training data were obtained. The training set was trained, validated, and tested with semantic segmentation models at 70%, 15%, and 15%.

#### 2.4.2 Silique and stalk point clouds segmentation based on DGCNN

Dynamic Graph Convolutional Neural Network (DGCNN) is a model based on Graph Convolutional Neural Network (GCN). It is specifically used for tasks such as image classification, point cloud classification and semantic segmentation. DGCNN uses local neighborhood information to perform feature extraction on each node. The global information is captured by a graph convolutional neural network to enable classification and segmentation tasks. It uses an integrated convolution module, EdgeConv, as its core, which models the points in the point clouds by using a graph approach. This enables the network to learn both local and global features of the point clouds while also learning the independent information of each point (Liu et al., 2022). Traditional 2D image convolution defines the local region of pixels using the size of the convolution kernel. EdgeConv, on the other hand, constructs a local region using a k-nearest neighbor (kNN) graph and performs convolution operations on it.

Since 3D point clouds are unstructured and unordered, most deep learning methods for processing 3D data in point clouds segmentation tasks convert the point clouds into a collection of sequential images or a voxel-based 3D data representation. However, multi-view and voxel-based representations can lead to unnecessary data redundancy and limit output resolution. Subsequently, PointNet (Qi et al., 2017a) directly processes 3D point clouds as deep neural network input data, but only based on global features of the point clouds, lacking local features. Therefore, PointNet++ (Qi et al., 2017b) proposes grouping and layering the point clouds, using PointNet to capture both local and global information. However, this method lacks the association between points. The introduction of DGCNN, based on PointNet++, adds the relationship between points, making local information more prominent. Compared to traditional CNN models, DGCNN is able to handle unstructured data and capture the relationship between local and global features. DGCNN can also be used to classify and segment unordered point sets without considering the order in which the points are arranged. In addition, DGCNN also has better robustness and is more capable of handling noisy and incomplete data. For the segmentation of the entire rapeseed siliques, high-precision local information is required for the segmentation of the siliques and stems, which can be met by DGCNN. Therefore, in this paper, DGCNN is used for semantic segmentation of rapeseed images to obtain the point clouds of the siliques without the stems.

This network model architecture is used as a model architecture for classification (top branch) and segmentation (bottom branch). The classification model takes as input *n* points, computes an edge feature set of size *k* for each point at the EdgeConv layer, and aggregates within each feature set to compute the response of the EdgeConv counterpart of the corresponding point. The output features of the last EdgeConv layer are aggregated globally to form a one-dimensional global descriptor that is used to generate the classification scores for class c. The partitioning model extends the classification model by connecting the one-dimensional global descriptor to all EdgeConv outputs. The EdgeConv output (as a local descriptor). It outputs a classification score for each point for the p semantic labels. The point cloud transformation block aims to align the input point set to the typical space by applying an estimated 3 × 3 matrix. To estimate the 3 × 3 matrix, a tensor is used that connects the coordinates of each point with the difference in coordinates between its k neighbors. The EdgeConv module takes as input a tensor of shape *n* ×*f* and computes the edge features of each point by applying a multilayer perceptron (mlp) with the number of layer neurons defined as {a_1_, a_2_,…, a_n_}, and generating a shape tensor × a_n_ after the set of neighboring edge features.

Since the network provides the probability prediction for each point in each class, the maximum probability value of the class and the point clouds label are used together to calculate the loss during network training. The network parameters are trained and learned through backpropagation. During network training, point clouds are input in batches (batch size) to reduce their differences in type and geometry. The equation of loss calculation during the training process uses the classic cross-entropy loss function as shown in (3).


(3) LOSS(x,label)=−log(exp[x(label)]/∑jexp[x(j)])

where x is the output of the network, the label is the corresponding label and j is the order of the output.

**Figure 2:**
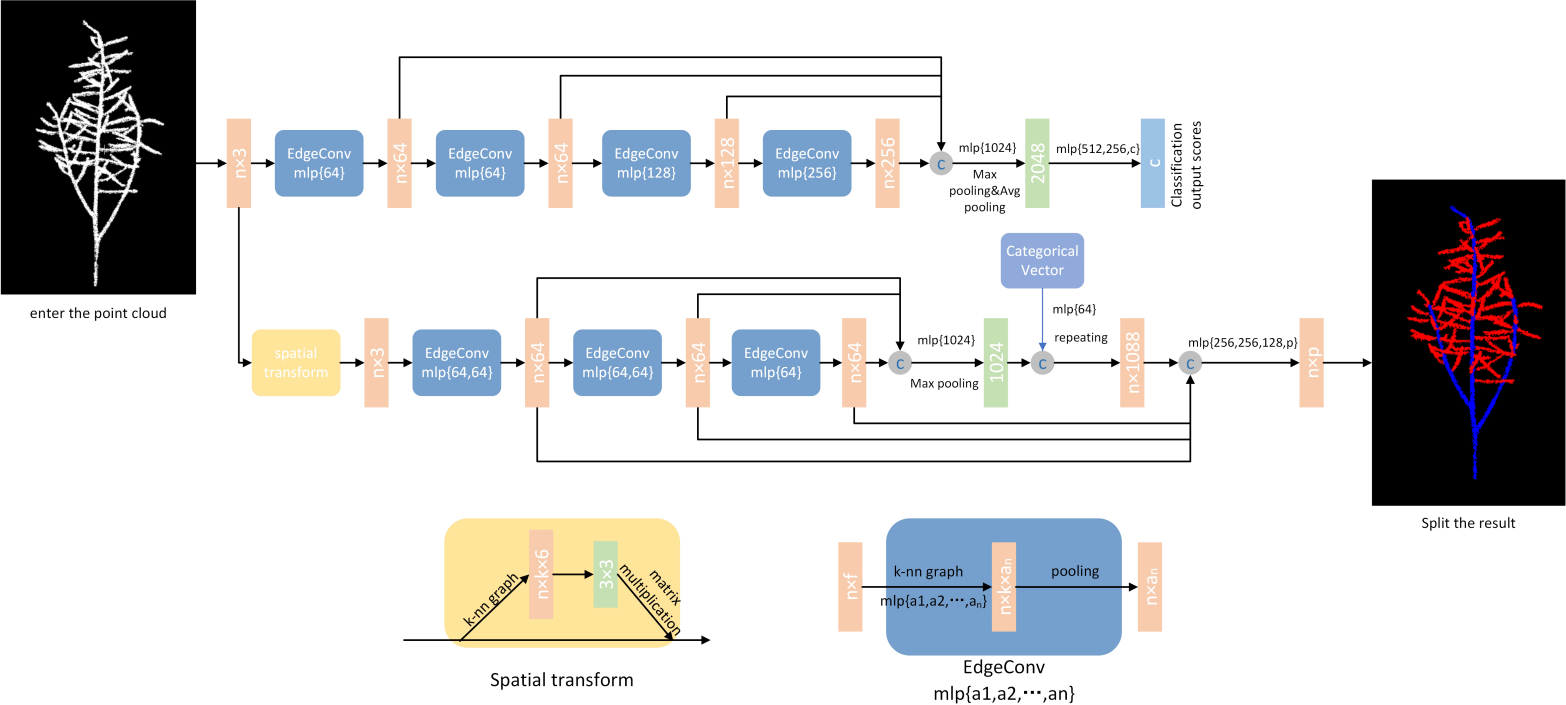
DGCNN network structure and segmentation effect. Model architectures: The model architectures used for classification (top branch) and segmentation (bottom branch). The classification model takes as input n points, calculates an edge feature set of size k for each point at an EdgeConv layer, and aggregates features within each set to compute EdgeConv responses for corresponding points. The output features of the last EdgeConv layer are aggregated globally to form an 1D global descriptor, which is used to generate classification scores for c classes. The segmentation model extends the classification model by concatenating the 1D global descriptor and all the EdgeConv outputs (serving as local descriptors) for each point. It outputs per-point classification scores for p semantic labels. ^©^: concatenation. Point cloud transform block: The point cloud transform block is designed to align an input point set to a canonical space by applying an estimated 3×3 matrix. To estimate the 3×3 matrix, a tensor concatenating the coordinates of each point and the coordinate differences between its k neighboring points is used. EdgeConv block: The EdgeConv block takes as input a tensor of shape n×f, computes edge features for each point by applying a multi-layer perceptron(mlp) with the number of layer neurons defined as {a_1_, a_2_,…, a_n_}, and generates a tensor of shape n×a_n_ after pooling among neighboring edge features.

During the training process, the network needs to learn how to set parameters based on the results of the loss function calculation in each epoch, which is directly influenced by the manually labeled data. The hyperparameters that need to be manually set in the network (parameters that cannot be learned) control the speed of convergence of the network’s loss and the training effect. This is because the more the loss calculation results converge, the better the semantic segmentation effect of the network. The hyperparameter settings in this article mainly focus on the optimizer, learning rate, training epochs, and batch size of inputs in each epoch. The optimizer used is Adam; the learning rate is set to 0.001; the training epoch value is set to 200; and the batch size is set to 20. The new point cloud data is segmented using a trained DGCNN network. The segmentation process consists of two processes: forward propagation and backward propagation. The forward propagation process is to input the point cloud data into the DGCNN network and get the output result of the network. The backward propagation process is to calculate the gradient of the loss function based on the network output result. The back propagation algorithm is used to update the network parameters, thus enabling the network to segment the point cloud data better. These parameters represent the maximum computational capacity supported by the computer during experimentation. The segmentation results are shown in **Figure 2**.

#### 2.4.3 Kd-tree radius searches - a segmentation method for sparse-dense point clouds mapping

Point clouds processing requires extremely high computational power. The DGCNN can effectively segment rapeseed point clouds. However, the computer used in this study is unable to process point clouds with more than 16,384 points. To handle large rapeseed point clouds, a new method of sparse-dense point clouds mapping is needed to solve the bottleneck caused by insufficient computational power. This method, based on the sparse crown siliques obtained by DGCNN segmentation, uses Kd-tree radius search to obtain the original dense point clouds of crown siliques(Evangelou et al., 2021).

KdTree is a high-dimensional space indexing structure used to partition k-dimensional data space. Its essence is a binary search tree with constraints. Approximate search algorithms based on Kd-Tree can quickly and accurately find the nearest neighbors of a search point, which is often used in feature point matching based on similarity (Xiao et al., 2022). There are two basic methods for similarity search algorithms in index structures: one is radius searches, and the other is K-neighbor searches (Zhang et al., 2020). Radius searches means finding all data in the dataset that is within the given search distance threshold (with the search point as the center and the search distance as the radius) that is less than the threshold distance from the search point (data within the radius). K-nearest neighbor searches is finding the K closest data points to the search point from the dataset. When K=1, it becomes the nearest neighbor searches. For 3D point clouds, all K-D trees are 3D. Building a k-d tree is a recursively unfolding process: at each level of expansion, all remaining datasets are divided along a specific dimension using a hyperplane perpendicular to the corresponding axis. At the root of the Kd-tree, all data is split according to the first dimension. The next level in the Kd-tree is divided along the next dimension. When all other dimensions are exhausted, it returns to the first dimension. The most efficient way to construct a K-D tree is to use a partitioning method similar to quicksort, placing the median at the root node, then placing values smaller than the median in the left subtree and values larger than the median in the right subtree, and finally repeating this process on the left and right subtrees until the last element is partitioned.

**Figure 3:**
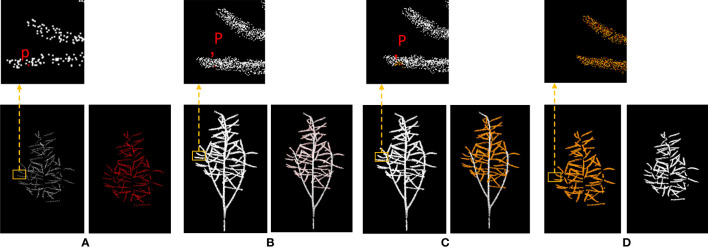
Diagram of the process of removing stalks from rapeseed silique. **(A)** Canopy silique point cloud after sampling is desired **(B)** Diagram of the original carob point cloud index process **(C)** Diagram of the search process for raw carob point clouds **(D)** Raw rapeseed canopy mirage point cloud.

**Figure 3** shows the process of removing the stems from rapeseed during the silique stage. The sparse crown siliques segmented by the DGCNN are shown in (a), while (b) shows the original point clouds of the entire rapeseed plant. The upper portion of (a) is a magnified view of the sparse crown siliques segmented by the DGCNN, while the upper portion of (b) shows the original point clouds of the entire rapeseed plant. In (a), a point is selected and its corresponding points in (b) is found. point clouds with a radius of 0.01 is then retained around this point. The retained point clouds are shown in yellow in (c). The final segmentation result is a dense crown silique, as shown in (d), which prepares for the accurate segmentation of individual silique in the subsequent steps.

### 2.5 Rapeseed silique identification based on the RANSAC algorithm

The contour of the reconstructed 3D point clouds of the rapeseed siliques is complete. Therefore, it is more suitable for line fitting identification. In this study, the plant point clouds is first segmented using the Euclidean clustering (Chen and Zhang, 2004). Then, based on the RANSAC algorithm (Barath et al., 2022), straight line fitting is applied to the clustered point clouds to segment the point clouds of the rapeseed siliques. Finally, the identification results are counted.

Plant point clouds exist in the form of multiple point clouds clusters in 3D space. In order to effectively detect and count the number of rapeseed siliques, it is necessary to determine the number of point clouds clusters and process each cluster separately to avoid missing or mis-segmenting the identification. Therefore, the plant point clouds are first segmented into multiple point clouds clusters using the distance-based Euclidean clustering algorithm, and then each cluster is processed separately. The point clouds clusters can be divided into two categories: those containing only one rapeseed silique point clouds, and those containing two or more rapeseed silique point clouds. Straight line segmentation is performed on each cluster. If the cluster contains rapeseed silique point clouds, the corresponding straight line can be fitted. Finally, the number of straight lines fitted for all point clouds clusters is counted to obtain the number of rapeseed siliques in the entire plant. A point clouds cluster may contain multiple rapeseed siliques, so the straight-line point clouds is fitted and segmented multiple times until the remaining un-fitted point clouds in the cluster is less than a certain value. Based on the measured width of the rapeseed siliques, the width range of the straight-line model is set to 0.03-0.05m, and the error threshold between the inliers and the model is set to 0.018m. At least 200 points are required to segment a straight-line model. During the straight-line fitting process, if the number of points in the point clouds is less than 200, the current model fitting result is regarded as a misidentification result. The straight-line model obtained in the previous fitting is retained as the identification result, and the current fitting result is discarded.

## 3 Experimental results and analysis

### 3.1 3D reconstruction experiments based on video and SFM

**Figure 4:**
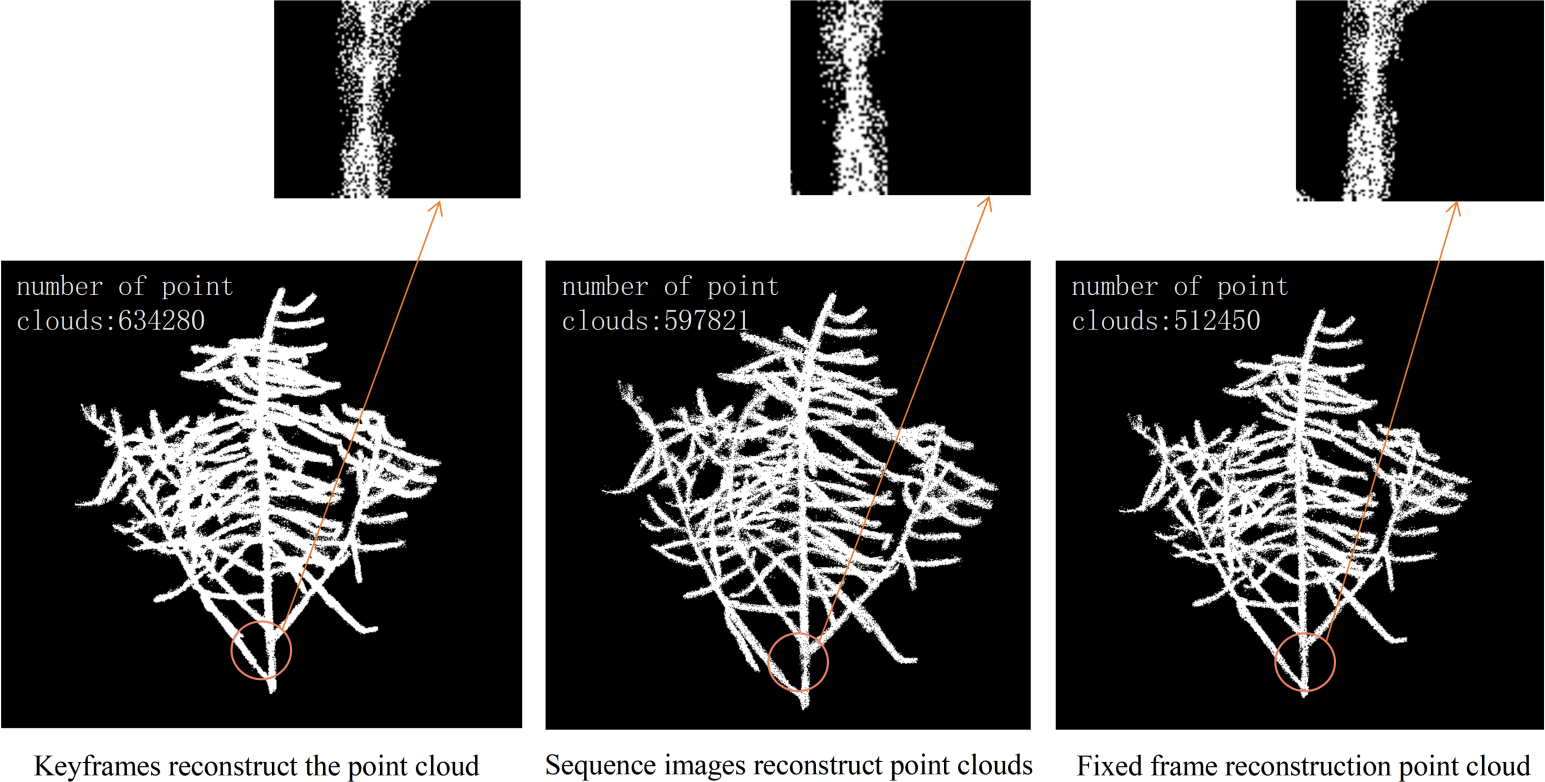
Comparison of point cloud quality obtained by three reconstruction methods.

An experiment was designed to compare three methods of 3D reconstruction: keyframe, sequence image, and fixed. Keyframes refer to the frames that capture the critical movements or changes of an object. Sequence images are the pictures taken around the rapeseed siliques, while fixed-frames are the video frames captured around the rapeseed siliques, with one frame extracted every ten frames. The experiment ensured that both sequence images and fixed-frames had 90 input images, while the keyframes were determined based on the algorithm results from the previous steps. The shooting method for sequence images was consistent with that of video frames. The quality of the reconstruction was compared from two aspects: the number of point clouds and the degree of restoration of details. That is, for the same rapeseed plant, the more point clouds, the more complete the structure and the fewer holes and missing parts. At the same time, the details of the point clouds, such as the siliques and stems, were observed, and the reconstruction effect of the details represented the quality of the point clouds. **Figure 4** shows three point clouds obtained 3D reconstruction using keyframe, sequence image and fixed frame, respectively, from left to right. The small images below **Figure 4** are an enlarged display of the circled part, which facilitates a more intuitive analysis of the reconstruction quality. Upon observing the enlarged part, it was found that the three methods had different reconstruction degrees of the 3D stem structure, with the keyframe reconstructing the most complete stem details, followed by the sequence image, and the fixed frame having the worst reconstruction. Through the analysis and comparison of point clouds obtained from multiple 3D reconstruction, it was found that the algorithm proposed in this paper had significant improvements compared to 3D reconstruction based on fixed frame, which could greatly improve the accuracy of subsequent phenotypic measurements of rapeseed siliques.

**Figure 5:**
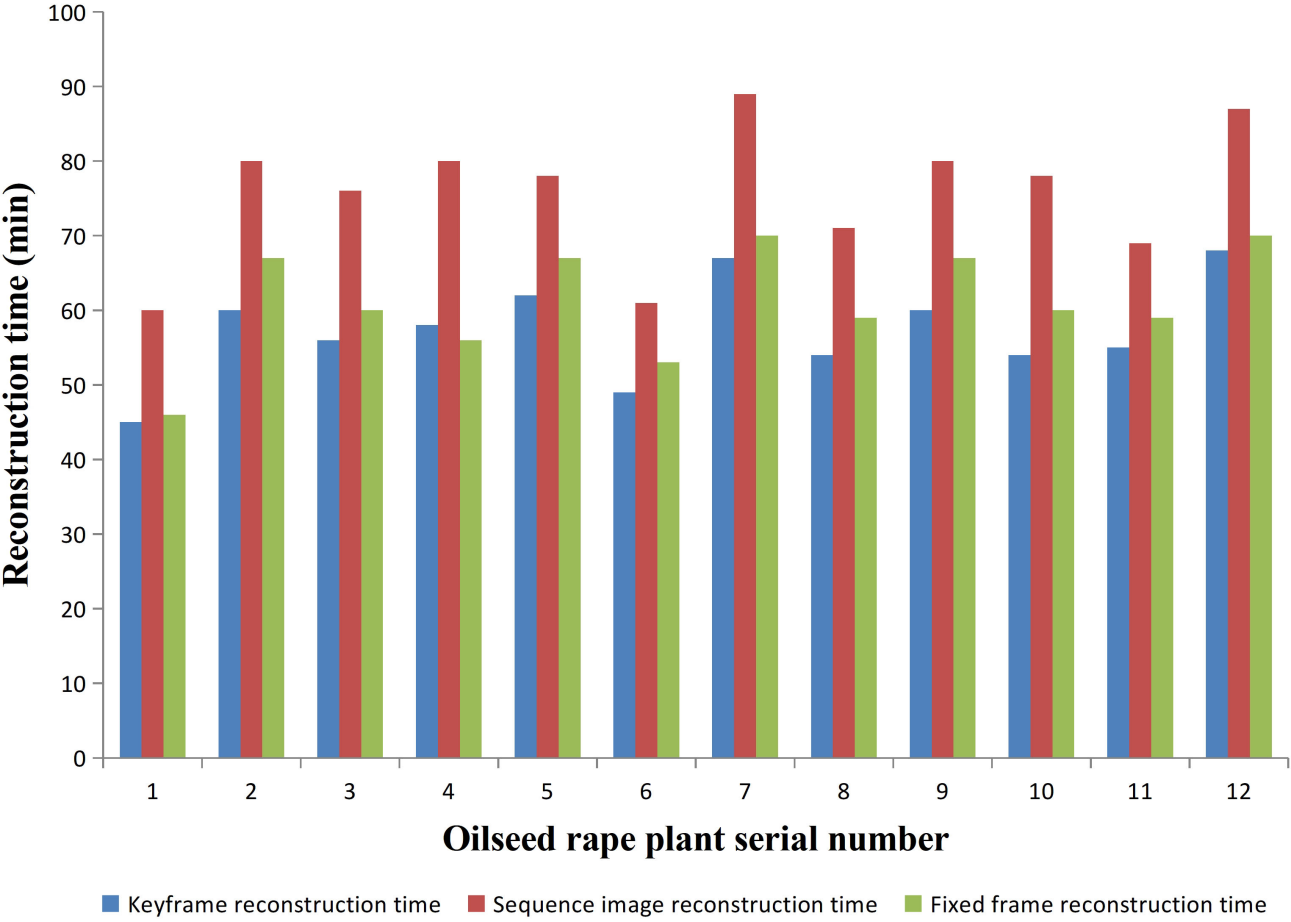
Comparison of reconstruction time of three reconstruction methods.

We conducted experiments on 12 rapeseed plants from three different varieties. The required time for 3D reconstruction based on the three methods was recorded and compared. The comparison of time consumption for these three methods is shown in **Figure 5**. For the same rapeseed plant, the duration of sequence image-based 3D reconstruction was significantly higher than that of fixed-frame and keyframe. 3D reconstruction based on fixed frame and keyframe, had similar durations, but keyframe-based 3D reconstruction was slightly shorter than fixed-frame-based reconstruction. Fixed-frame-based reconstruction improved the efficiency of reconstruction by 19.39% compared to sequence image-based reconstruction, while keyframe-based reconstruction improved efficiency by 24.4% compared to sequence image-based reconstruction. Therefore, if judged solely on the basis of the reconstruction time, sequence image-based reconstruction took the longest time, followed by fixed-frame-based reconstruction, and keyframe-based reconstruction took the shortest time.

### 3.2 Experimental segmentation of silique point clouds

**Figure 6:**
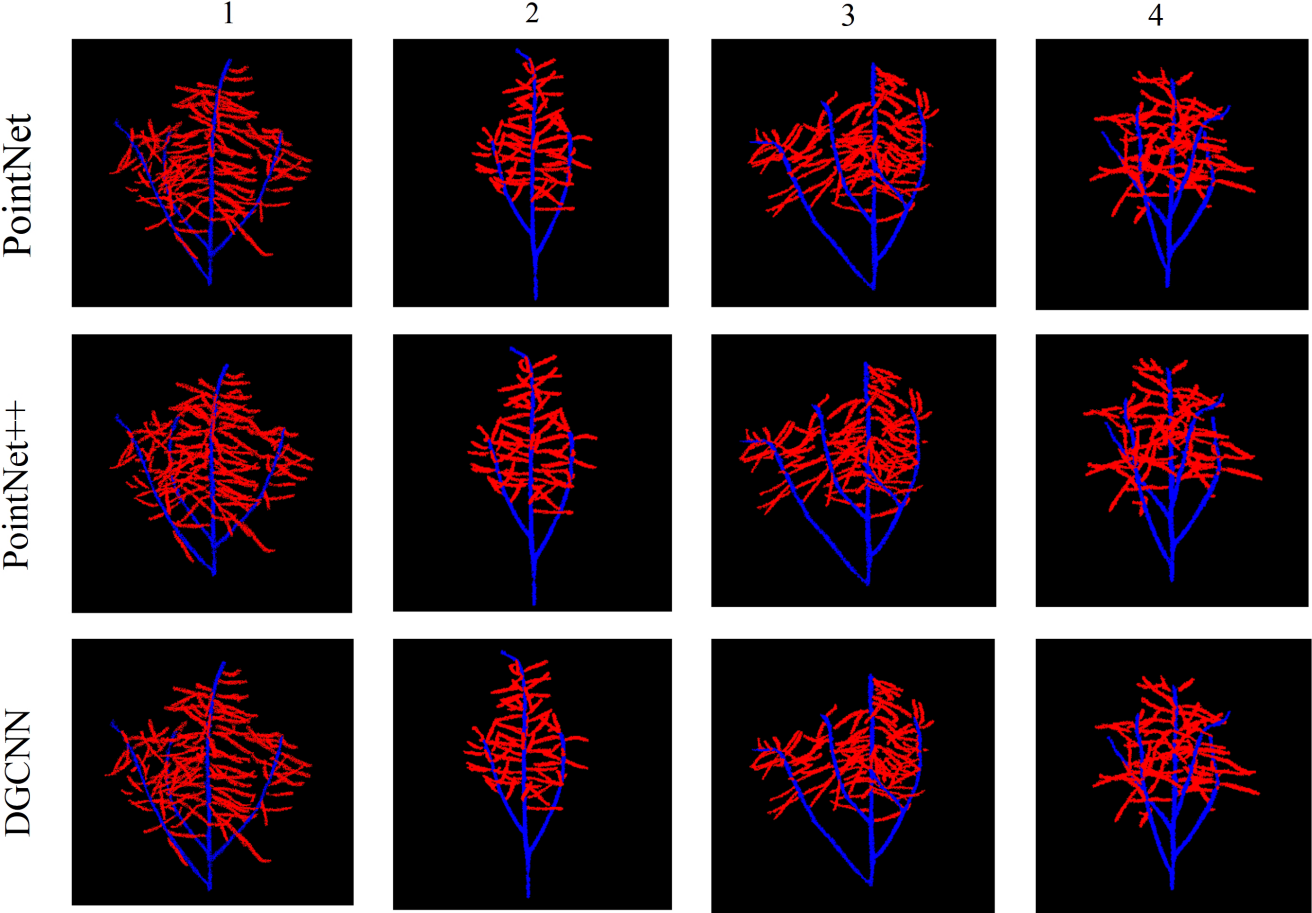
Renderings of three types of network segmentation.

Semantic segmentation based on deep learning has a very broad development prospect in the field of computer vision. However, many network models with good segmentation results occupy a large amount of memory and take a long time to process 3D point clouds (Mirande et al., 2022). Based on the DGCNN-sparse-dense point clouds mapping, it has faster processing speed and better segmentation results, and consumes less memory. We conducted experiments on four rapeseed siliques. **Figure 6** shows the segmentation results of PointNet, PointNet++, and DGCNN for the four rapeseed siliques, respectively.

**Table 1** shows a quantitative comparison of the three networks. DGCNN achieved the best results in most cases and outperformed other networks in all four average quantization metrics.

**Table 1:** The semantic segmentation performance of the three networks was quantitatively compared.

	Methods	1	2	3	4
Prec (%)	PointNet	77.16	93.56	89.18	96.61
	PointNet++	87.79	95.91	91.71	96.70
	DGCNN	**92.72**	**96.37**	**92.39**	**97.97**
Rec (%)	PointNet	91.21	89.08	86.89	98.28
	PointNet++	90.84	83.96	91.94	97.82
	DGCNN	**97.7**	**89.55**	**93.32**	**98.21**
F1 (%)	PointNet	93.67	86.87	87.87	97.76
	PointNet++	94.84	91.92	91.82	97.79
	DGCNN	**97.25**	**93.86**	**94.54**	**98.31**
IoU (%)	PointNet	88.09	92.42	93.42	93.26
	PointNet++	90.18	96.45	96.65	95.51
	DGCNN	**94.35**	**95.68**	**96.77**	**96.74**

The reconstructed rapeseed silique point clouds were manually segmented into rapeseed stems and canopy siliques using CloudCompare software. The segmented point clouds were down sampled to 4096, 8192, 12288, 16384, and 18432 points for each rapeseed siliques. Due to the large number and complex structure of the rapeseed silique point clouds, down sampling to 4096 points not only reduced the number of point clouds too much but also destroyed the structure. When the number of point clouds is too small, it cannot be restored to the original number of point clouds under the method of sparse-dense point clouds mapping, which leads to the inability to recognize subsequent siliques. On the other hand, down sampling to 18432 requires a lot of resources, making it impossible for the computer to run. **Table 2** shows that when down sampled to 8192 point clouds, the training and processing time is the shortest and the siliques recognition rate is the highest after sparse-dense point clouds mapping.

**Table 2:** Quantitative comparison of silique down sampling to different point cloud numbers.

The number of point clouds after down sampling	4096	8192	12288	16384	18432
Times(h)	1.25	2.5	3.75	5	×
The number of point clouds after restoration	×	250241	272783	301013	×
Silique recognition rate	×	98.12%	91.99%	98.03%	×

### 3.3 Experimental recognition of silique point clouds

**Figure 7:**
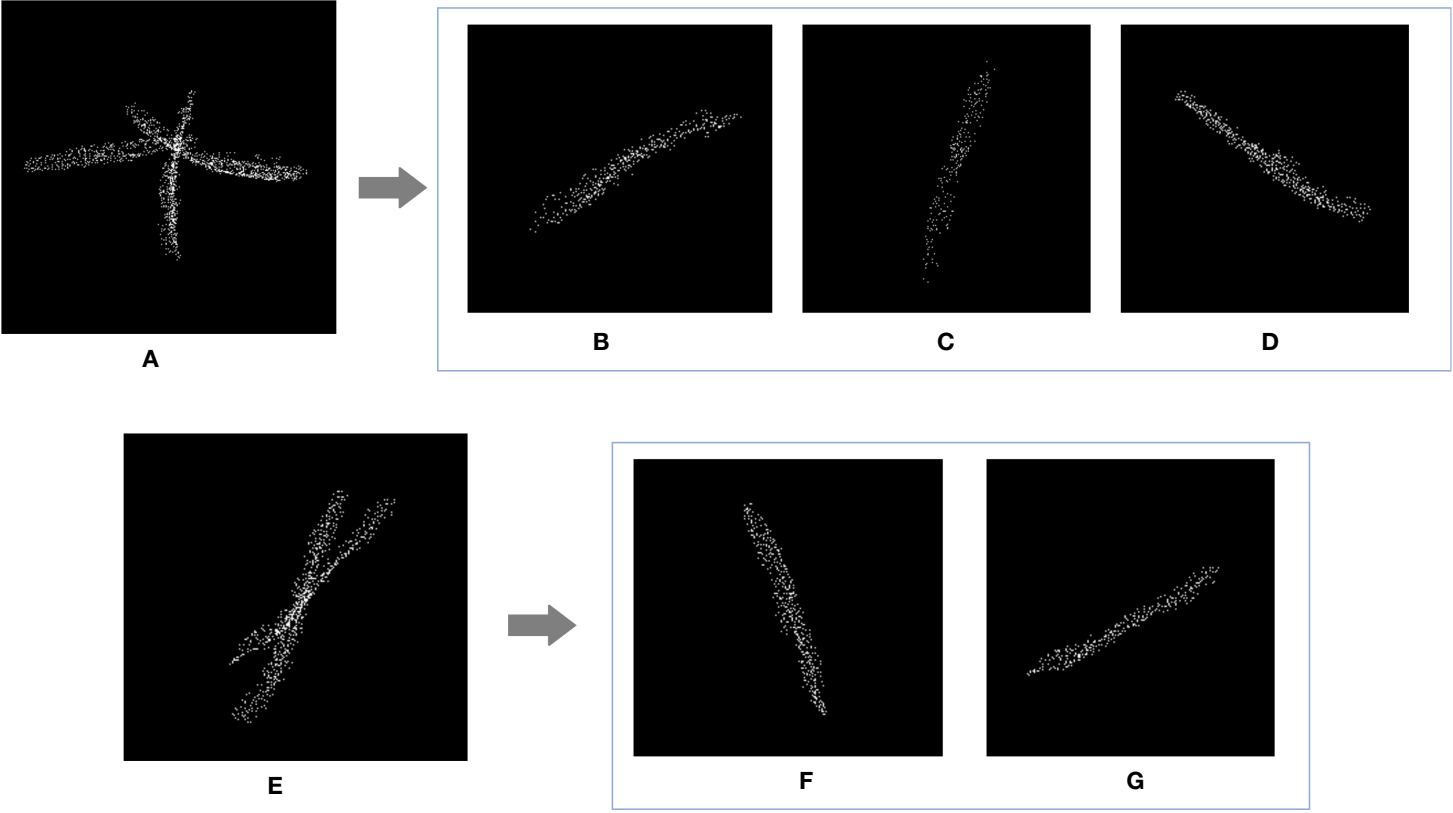
Rapeseed silique segmentation results. **(A)** Diagram of three siliques adhesions **(E)** Diagram of two siliques adhesions **(B, C, D, F, G)** Single silique.

The recognition results were compared with the ground truth. As shown in **Table 3**, numbers 1-4 were Huayouza 62, numbers 5-8 were Zhongshuang 6, and numbers 9-12 were Dadi 55. The total recognition precision was 97.80%, the mean absolute percentage error was 1.96% and the R^2^ was 0.96. **Figure 7** shows the segmentation results of the siliques. **Figures 7A, E** show two different cases of siliques adhesion. **Figures 7C, B ,D, F, G** show the recognition results under the adjacent or adhesive state of the siliques. Even when the siliques were heavily adjoined and occluded, our method could distinguish and recognize different siliques. Our method segmented and recognized the siliques based on their spatial shape features. However, there were a small number of missed recognitions during the silique’s recognition process. The reasons for missed recognition were mainly due to the silique point clouds having too few points or irregular shapes, which did not meet the fitting conditions set by the RANSAC algorithm. When the siliques were extremely small in shape and had fewer point clouds after down sampling and statistical filtering, they could be easily treated as noise and removed. In addition, incomplete or irregular straight-line contours resulted in the inability to fit a straight line.

**Table 3:** Silique identification and counting results.

Rapeseed varieties	Plant number	True value	Correct identification number of siliques	Missing identification number of siliques	Pr(%)
Huayouza 62	1	126	124	2	98.41
	2	196	192	4	97.96
	3	110	108	2	98018
	4	119	117	2	98.32
Zhongshuang 6	5	104	102	2	98.08
	6	139	135	4	97.12
	7	85	83	2	97.65
	8	99	98	1	98.99
Dadi 55	9	129	126	3	97.67
	10	105	104	1	99.05
	11	124	121	3	97.58
	12	118	115	3	97.46
Total		1457	1425	32	97.80

## 4 Discussion

(1) In this paper the SFM method was chosen for the 3D reconstruction of rapeseed silique. The method is inexpensive and the quality of the 3D reconstruction is high, but the time consumption is huge. The time required for one 3D reconstruction was more than 30 minutes. Moreover, more images are required to obtain higher quality 3D point clouds, which leads to a dramatic increase in time consumption. Therefore, further research is needed to explore optimization methods for SFM, such as adding GPU acceleration.
(2) The point cloud segmentation method proposed in this paper requires down sampling of whole rapeseed plant point cloud. As can be seen from the above experiments, due to the large number of point clouds and complex structure of the whole rapeseed silique. When down sampling to 4096, not only the number of point clouds is too small, but also the structure is destroyed. When the number of point clouds is too small, it cannot be restored to the original number of point clouds under the sparse-dense point cloud mapping method. This in turn results in the subsequent number of siliques not being identified. And when down sampling 18432, a large number of resources are required causing the computer to become inoperable. Therefore, the range for down sampling the whole rapeseed silique point cloud is 4096-18432.
(3) The Euclidean clustering-RANSAC segmentation and identification method has certain limitations in the segmentation of the siliques point cloud identification. The number of siliques identified by this method is often slightly smaller than the actual number. There are three main reasons for this. Firstly, in the image pre-processing process, some small siliques are mistakenly rejected. Secondly, in the point cloud filtering session, some siliques were broken into small pieces and mistakenly considered as outlier noise and were rejected. Thirdly, the parameter settings for segmenting the point cloud of rapeseed silique based on the RANSAC algorithm for linear fitting of the clustered point cloud. If the width range of the line model is set to less than 0.03m or greater than 0.05m, it can cause changes in the siliques shape and inaccurate counting.

## 5 Conclusion

This paper provides a process and methodology that can be used as a reference for segmenting dense plant point clouds with complex structures. We take the dense point clouds of whole rapeseed plants at the siliqua stage with complex morphological features as a typical example. The DGCNN used in this paper performs semantic segmentation on the entire rapeseed point clouds. Compared with instance segmentation, the data annotation cost of semantic segmentation is much lower. Moreover, the DGCNN adds relationships between points on the basis of PointNet++, making local information more prominent. For the entire rapeseed at the siliqua stage, high-precision local information is required for the segmentation between siliqua and stem. DGCNN is well suited for this task. After segmentation, the sparse canopy siliqua point clouds without stem are obtained, and then the sparse-dense point clouds mapping segmentation method is used to segment the original rapeseed canopy siliqua point clouds. This method greatly reduces the computational requirements for deep learning network point clouds segmentation. Targeted solutions are proposed for several difficulties in identifying mature rapeseed siliqua.

(1) This paper proposes a similarity-based video keyframe extraction algorithm. The algorithm effectively removes redundant and motion-blurred images, saving reconstruction time and cost, and ultimately ensuring a more complete view of the extracted video frames. Image enhancement processing of video frames improves contrast and enhances edge details, thereby improving the quality of the 3D model.
(2) To solve the problem of recognizing and counting the overall number of rapeseed siliques, this paper proposes a method based on DGCNN-sparse-dense point clouds mapping to segment the crown siliques. This method can remove the stems of the entire rapeseed siliques while maintaining complete spatial information, compared to only using DGCNN network to segment sparse crown siliques. This method not only improves the accuracy of subsequent siliques point clouds recognition and counting, but also greatly reduces the algorithm’s requirements for computer computing power.
(3) For the crown siliques, this paper uses Euclidean clustering segmentation and a line fitting method based on random sample consensus algorithm to segment and recognize the siliques. Based on the contour features of line shapes, this method can greatly improve the accuracy of siliques recognition, which is of great significance for yield estimation and subsequent cultivation.
(4) This paper identifies 1457 siliques from 12 rapeseed plants, with a total identification accuracy rate of 97.80%. When comparing the total calculated number of siliques with the actual value, the coefficient of determination is 0.97, and the average absolute percentage error is 1.96%. This method can effectively recognize adhered siliques, as well as identify and count the entire siliques. The proposed method not only has extremely low cost, good portability, and high precision, but also can effectively save runtime and improve efficiency, greatly improving the accuracy of siliques counting.

## Data availability statement

The original contributions presented in the study are included in the article/supplementary material . Further inquiries can be directed to the corresponding author.

## Author contributions

YQ: Conceptualization, lnvestigation, Methodology, Visualization, Writing-original draft. MZ: Supervision, Software. ZH: Data curation. SW: Data curation. BH: Formal analysis. CP: Validation. QL: Funding acquisition, Supervision, Resources. SX: Methodology, Investigation, Supervision,Writing-review editing. GZ: Funding acquisition. All authors have read and agreed to the published version of the manuscript.
